# Of causes and symptoms: using monitoring data and expert knowledge to diagnose the causes of stream degradation

**DOI:** 10.1007/s10661-023-11741-5

**Published:** 2023-09-28

**Authors:** Katharina Rettig, Renate Semmler-Elpers, Denise Brettschneider, Daniel Hering, Christian K. Feld

**Affiliations:** 1 Faculty of Biology, Aquatic Ecology, University of Duisburg-Essen, Universitätsstr. 5, Essen, 45141 Germany; 2 State Agency for the Environment Baden-Württemberg, Griesbachstr. 1, Karlsruhe, 76185 Germany; 3 Center for Water and Environmental Research, University of Duisburg-Essen, Universitätsstr. 2, Essen, 45141 Germany

**Keywords:** Stream and river management, Biological metrics, Multiple stressors, Diagnostic approach, Data synthesis

## Abstract

Ecological status assessment under the European Water Framework Directive (WFD) often integrates the impact of multiple stressors into a single index value. This hampers the identification of individual stressors being responsible for status deterioration. As a consequence, management measures are often disentangled from assessment results. To close this gap and to support river basin managers in the diagnosis of stressors, we linked numerous macroinvertebrate assessment metrics and one diatom index with potential causes of ecological deterioration through Bayesian belief networks (BBNs). The BBNs were informed by WFD monitoring data as well as regular consultation with experts and allow to estimate the probabilities of individual degradation causes based upon a selection of biological metrics. Macroinvertebrate metrics were shown to be stronger linked to hydromorphological conditions and land use than to water quality-related parameters (e.g., thermal and nutrient pollution). The modeled probabilities also allow to order the potential causes of degradation hierarchically. The comparison of assessment metrics showed that compositional and trait-based community metrics performed equally well in the diagnosis. The testing of the BBNs by experts resulted in an agreement between model output and expert opinion of 17–92% for individual stressors. Overall, the expert-based validation confirmed a good diagnostic potential of the BBNs; on average 80% of the diagnosed causes were in agreement with expert judgement. We conclude that diagnostic BBNs can assist the identification of causes of stream and river degradation and thereby inform the derivation of appropriate management decisions.

**Supplementary Information:**

The online version contains supplementary material available at 10.1007/s10661-023-11741-5.

## Introduction

Europe’s running waters are largely degraded. In 2015, 56.1% of Europe’s streams and rivers failed to achieve a good ecological status or potential (EEA, 2023). The reasons for this failure are manifold and include morphological and hydrological pressures, nutrient enrichment from diffuse sources, and chemical pollution (Lemm et al., 2021), which may also interact in complex ways. According to the European Water Framework Directive (WFD; Directive 2000/60/EC), ecological status assessment is primarily based on biological quality elements (BQEs), comprising fish, macroinvertebrates, higher plants, and algae, and supported by physicochemical and hydromorphological parameters. The BQEs are usually quantified as community metrics (e.g., number of taxa, proportion of feeding types, or habitat preferences) based on taxalists (Birk et al., 2012). These metrics are known to respond to a variety of anthropogenic stressors (Birk et al., 2012; Hering et al., 2010) and thus are used to integrate the overall impact of stressors into an ecological status class ranging from 1 (high) to 5 (bad). However, many metrics respond unspecific to individual stressors and rather integrate their impacts across spatial and temporal scales (e.g., Hering et al., 2006; Laini et al., 2018; Lemm et al., 2019). This integrative nature of assessment metrics is desirable, because the final ecological status of a river water body has to reflect the overall impact of all stressors affecting the biota in that water body. However, as soon as management measures need to be defined to improve the ecological status, many assessment metrics bear only little information about the causal stressor(s) behind a moderate or worse ecological status. The metrics do not allow to disentangle the individual stressors’ effects, which is a prerequisite for developing targeted measures (Gieswein et al., 2017). Consequently, the diagnosis of the causes of stream degradation merely from biological monitoring data constitutes a major challenge for river managers.

To overcome this obstacle, several approaches for stressor identification have been developed. With CADDIS (U.S. EPA, 2017) and Eco Evidence (Nichols et al., 2011) online applications have been implemented in the U.S. and Australia that allow users to evaluate apparent cause-and-effect relationships and identify likely causes of aquatic system degradation based on evidence. Evidence may be extracted from, inter alia, literature, experiments, predictions, data, or qualitative opinion. In order to complement current assessment and management practices for streams and rivers within the EU, some studies have developed purely data-driven diagnostic approaches based on regular monitoring data. Mondy and Usseglio-Polatera (2013) developed random forest (RF) models based on macroinvertebrate traits to disentangle several pressure categories and calculate their impairment probabilities. Similar to this approach, Dézerald et al. (2020) used fish metrics as predictors in RF models to examine pressure patterns and identify the most influential pressures. In addition, Clews and Ormerod (2009) achieved an improved diagnostic capability by combining several standard biological indices, while Baattrup-Pedersen et al. (2016) and Statzner and Bêche (2010) used (multiple) functional traits of macroinvertebrate taxa, such as small size and flattened body, to withstand stressful flows, to discriminate between stressors.

Against this background, the discriminatory diagnosis of individual stressors could be improved by approaches that allow for the combination of multiple biological metrics as well as the integration of different types of evidence (Feld et al., 2020). Probabilistic models such as Bayesian belief networks (BBNs) provide a suitable framework to integrate evidence from monitoring data with evidence from literature and knowledge of experts (Feld et al., 2020; Trigg et al., 2000). A BBN graphically represents dependencies between variables, while the dependencies are expressed as conditional probabilities (Jensen & Nielsen, 2007). The main advantages of BBNs include the capability to synthesize knowledge (McCann et al., 2006) and to quantify the uncertainty that is associated with the model outcome (Uusitalo, 2007). Recent applications of BBNs include the diagnosis and ranking of potential causes of degradation using biological metrics (Feld et al., 2020). This approach is similar to medical diagnosis (Elosegi et al., 2017), where symptoms (biological metrics) are related to potential causes (of diseases). Besides this example, however, the utility of probabilistic models such as BBNs for river ecosystem diagnosis is still in its infancy—although the monitoring data from more than 100,000 river water bodies and several monitoring cycles since 2005 (EEA, 2023) provides an excellent database for the development of river diagnostic tools as suggested by Feld et al. (2020) and shown by Dézerald et al. (2020). The data has been gathered by numerous experts, including local river basin managers, biologists, and practitioners, whose invaluable knowledge could also help disentangle the responses of stream biota to multiple stressors. Yet, to date, these invaluable assets remain largely unexploited.

Here, we present an approach that probabilistically links anthropogenic stressors (potential degradation causes) with biological responses (macroinvertebrate and diatom metrics) for three groups of stream types in the Federal State of Baden-Württemberg (Germany). For each of these groups, a BBN was developed to provide the probabilistic model framework. We hypothesized that the simultaneous utility of numerous biological metrics allows for a discrimination of individual causes, even in the absence of stressor-specific metrics. We further hypothesized that metrics reliably indicating individual stressors are applicable across different stream types or even eco-regions. The diagnostic BBNs were validated and tested against external data and the expertise of local experts. To our knowledge, this is the first study to present twofold-validated diagnostic BBNs for running waters. The final integration of the BBNs into web-based diagnostic tools allows an end user to easily identify potentially causal stressors and their hierarchy within a targeted running water body.

## Materials and methods

### Study area

We used WFD monitoring data from 783 stream and river monitoring sites in the Federal State of Baden-Württemberg (Germany). Each site is assigned to a stream type (stream typology according to Pottgiesser, 2018). The stream types are characterized by four criteria, namely, ecoregion (according to Illies, 1978), elevation, size of catchment area, and catchment geology. For the purpose of this analysis, they were assigned to one of three groups based on their similarities: ‘streams of the low mountain ranges,’ ‘rivers of the low mountain ranges,’ and ‘streams/rivers of the pre-alpine region’ (Table 1).

**Table 1:** Grouping of stream types. Monitoring sites refer to macroinvertebrate monitoring

**Group of stream types**	**Types**	**Monitoring sites**
Streams of the low mountain ranges	5: coarse substrate-dominated, siliceous mountainous streams 5.1: fine substrate-dominated, siliceous mountainous streams 6: coarse substrate-dominated, carbonaceous mountainous streams 7: fine substrate-dominated, carbonaceous mountainous streams	426
Streams/rivers of the pre-alpine region	2.1: streams of the pre-alpine region 2.2: small rivers of the pre-alpine region 3.1: streams of the young moraines of the pre-alpine region 3.2: small rivers of the young moraines of the pre-alpine region	121
Rivers of the low mountain ranges	9: siliceous, fine to coarse substrate-dominated mountainous rivers 9.1: carbonaceous, fine to coarse substrate-dominated mountainous rivers 9.2: large mountainous rivers	246

### Biological data

The State Agency for the Environment of Baden-Württemberg provided data on macroinvertebrates and diatoms that were part of the regular WFD monitoring. Macroinvertebrates were sampled at 783 sites between 2010 and 2016 using a multi-habitat sampling protocol (Meier et al., 2006a) and identified in the laboratory to species level (except for Oligochaeta and Diptera: family level) to obtain the taxonomic level given by an operational taxalists (Haase & Sundermann, 2004). The taxalists were first processed with ASTERICS v.4.04 (https://gewaesser-bewertung.de/index.php?article_id=419) and later with PERLODES v.5.0 (https://www.gewaesser-bewertung-berechnung.de/index.php/perlodes-online.html), which allows for the calculation of more than 300 macroinvertebrate community metrics, including measures of abundance, biodiversity, community, and functional composition. Based on the author’s expertise, 32 macroinvertebrate metrics were pre-selected.

For 114 of the 783 monitoring sites, results of the Trophic Diatom Index, an indicator of the trophic status, were provided. Benthic diatoms were sampled under WFD monitoring programs in 2013 according to the protocol described in Schaumburg et al. (2012) and determined to species level whenever possible. The resulting taxalists were processed with Phylib v.6.0 (https://gewaesser-bewertung-berechnung.de/index.php/phylib-online.html).

### Environmental data

The State Agency for the Environment of Baden-Württemberg also provided data on various potential stressors. For each monitoring site, data on catchment area and percentages of land use types within the catchment for the year 2009, including urban area, agriculture, special crops, intensive grassland, and coniferous forest, were provided. Hydromorphological conditions were recorded on site, similar to the descriptions in Gellert et al. (2014), between 2010 and 2016 by mapping 18 structural parameters along 100-m segments, summarized in the six main parameters (1) channel evolution, (2) longitudinal profile, (3) cross profile, (4) riverbed structure, (5) bank structure, and (6) floodplain condition. Subsequently, the recorded conditions were compared to reference conditions, and depending on the deviation, each parameter was assessed on a scale ranging from unchanged (class 1) to completely modified (class 7). These assessment results were comprised in a shapefile of the stream and river network of Baden-Württemberg. For each monitoring site, we computed upstream stretches of 1 km and 5 km in ArcView v.3.3 (ESRI, 2002). Hydromorphology assessment data within these stretches, as well as at the location of each monitoring site, were extracted from the shape file in ArcMap Desktop v.10.8 (ESRI, 2020). Subsequently, we calculated the weighted means of the hydromorphological assessment classes within these stretches.

To spatially match the different data sources, the closest upstream gauging stations were assigned to the monitoring sites in ArcMap Desktop v.10.8 (ESRI, 2020). From the gauging data, regionalized runoff values (1981–2010), including extreme and reoccurring flood events (m^3^/s), as well as the ratio of mean high and low water flow, were derived. Such a network analysis was also applied to identify the closest chemical monitoring station as well as the closest and all sewage treatment plant upstream of each monitoring site. Annual means of the plant parameters NO_3_-N (mg/L) and o-PO_4_-P (mg/L) at outlet, as well as maxima of pH values, were calculated for the year before macroinvertebrate sampling. Network analysis was also used to identify if a monitoring site is located in a backwater, based on shape file of location and length of backwaters for the year 2019. Fine sediment coverage was estimated using the field protocol of the macroinvertebrate sampling. We calculated the fine sediment proportion at a monitoring site as the sum of percentages of psammal (sand), argyllal (loam), pelal (sludge), and FPOM. For the water quality parameters BOD_5_ (mg/L), NO_2_-H (mg/L), and NH_4_-N (mg/L), Cl (mg/L), SO_4_ (mg/L), and conductivity (µS/cm), the annual mean of all measurements in the year before macroinvertebrate sampling was calculated. For O_2_ (mg/L), the minimum of all measurements in the year before sampling was calculated.

Overall, a total of 81 environmental variables that could potentially act as stressors were identified. As not all data were available for each monitoring site, the coverage of the individual parameters ranged from 113 to 765 of the 783 monitoring sites, with data on chemical parameters showing the lowest coverage and hydromorphological conditions the highest.

### Relationships between stressors and metrics

**Fig. 1:**
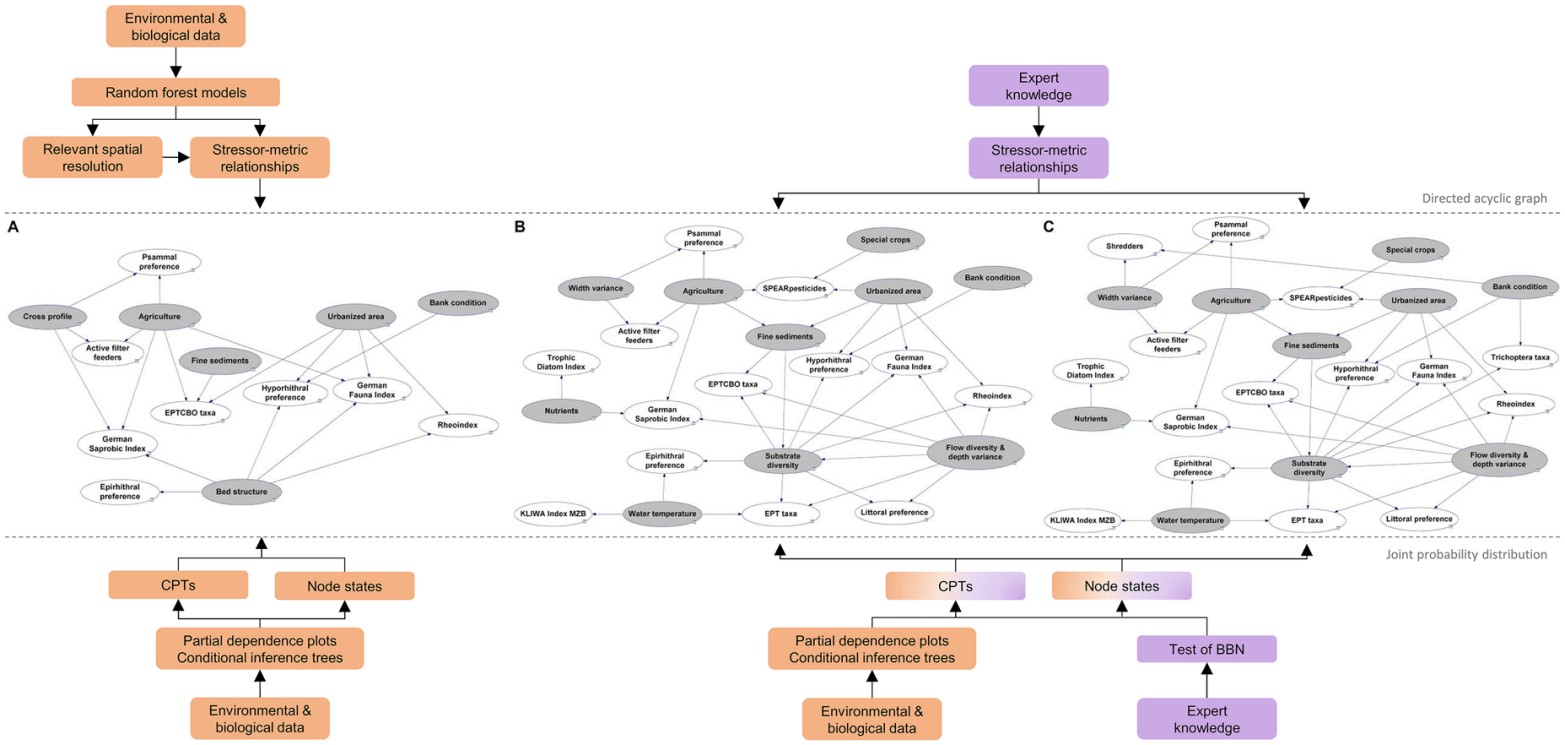
Development of the BBN structure for the group ‘streams of the low mountain ranges’ (SLMR), including crucial steps of integrating monitoring data (orange) and expert knowledge (purple). The initial, only data based, BBN (**A**) was revised during an expert workshop. Experts’ feedback and subsequent additional data analyses resulted in an intermediate BBN (**B**). The final BBN (**C**) resulted from some additional suggestions from and two testing rounds by experts. Degradation causes in gray, biological metrics in white

All analyses underlying the development of the BBNs were performed in R v.4.1.0 (R Core Team, 2021). To exclude redundant hydromorphological parameters, the parameters were tested for collinearity by calculating correlation coefficients (Pearson) with the ade4 package (Dray & Dufour, 2007). If the Spearman rank correlation coefficient of a pair was greater than 0.8, one of the two parameters was excluded from further analyses. The identification of the spatial scale at which the remaining hydromorphological parameters show the strongest influence on biota and the selection of potentially diagnostically useful stressor-metric relationships were done using the randomforestSRC package (Ishwaran & Kogalur, 2007) (Fig. 1). RF models can handle incomplete datasets with a large number of explanatory variables and are suitable for analyzing non-linear relationships (Breiman, 2001). The output includes the relative importance of each predictor variable to the model. Partial dependence plots (Hastie et al., 2009; Friedman, 2001) help to uncover individual cause-and-effect relationships.

In the course of the identification of the most influential spatial scale, the German Fauna Index and the share of Ephemeroptera, Plecoptera, and Trichoptera taxa (% EPT) were used as proxies for biota. Both are sensitive to hydromorphological conditions (Feld et al., 2014). A RF model was built for each of these metrics per group of stream types. Across all six RF models, hydromorphological parameters within a stretch of 5 km upstream of the monitoring sites predominantly showed the strongest relative influence to the deviance explained by the model. This resulted in the selection of 14 structural parameters and four main hydromorphological parameters at the identified most influential spatial scale.

The next step was to build RF models for each group of stream types for each pre-selected biological metric (response variable), using the 33 considered potential causes of degradation as predictor variables. Besides the hierarchy of importance of single predictor variables, partial dependence plots were visually examined for break points. Here, a break point is defined as a sharp increase or decrease in the value of the response variable at a certain value along the predictor’s gradient, in particular those that mark a shift from low to high values (or vice versa). Since visual detection bears the risk to be biased by the viewer’s judgement, conditional inference tree analyses were additionally performed with the partykit package (Hothorn et al., 2006), to align the visually identified break points. Conditional inference trees use *p*-values to split the distribution of a response variable along the gradient of predictor variables. The number of levels of predictor variables was restricted to three. The relationship between a potential stressor and a metric was only included in the initial BBN structure, if the stressor was ranked in the top quarter of the relative importance hierarchy and the partial dependence plot showed a sharp increase or decrease.

## BBN development

A BBN is a probabilistic model, which graphically represents relationships between predictors and response variables as nodes, which are connected by arrows pointing from predictor nodes (parent) to response nodes (child) (Jensen & Nielsen, 2007). In discrete BBNs, each node has a finite set of states representing a variable’s gradient. The probability of a child being in a particular state depends on the combination of states of its parents. This conditional dependency is captured in conditional probability tables (CPTs). The conditional probabilities can be informed by empirical data, evidence reported in literature, or knowledge of experts (McCann et al., 2006; Uusitalo, 2007). Furthermore, a probabilistic model inherently addresses the uncertainty and variability associated with the assignment of conditional probabilities (Jensen & Nielsen, 2007).

A discrete BBN was developed for each group of stream types in GeNIe Modeler v.2.5 (BayesFusion, LLC, 2020). Figure 1 graphically represents the development process of the BBN for the group ‘streams of the low mountain ranges.’ The initial BBNs were exclusively based on monitoring data. Break points of stressor–metric relationships, which were identified by the previously described analyses, were used to define the ranges of node states (Table S1–S3). For populating the models’ CPTs, we revisited the calculated conditional inference trees (“Relationships between stressors and metrics”), which display the distribution of values of a biological metric depending on the value of one or more potential degradation causes (for more details see Feld et al., 2020). If these analyses were not sufficient, the data distribution across the defined node states was inspected using box-and-whisker plots and partially complemented with the authors’ expertise.

Throughout the development of the BBNs, the involvement of experts from environmental authorities and spatial planning offices in Baden–Württemberg (Table S4) was an iterative process. The experts recommended several structural modifications of the BBNs: (1) considering individual hydromorphological parameters instead of aggregated main parameters (e.g., width variance instead of cross profile) and (2) including the KLIWA-Index_MZB_ and the Trophic Diatom Index for a better approximation of warming and nutrient enrichment, respectively. The former index has been developed to indicate warming effects (Sundermann et al., 2022), whereas the latter index is strongly linked to eutrophication (Schaumburg et al., 2012). The experts also suggested additional stressor-metric relationships and revised existing ones. The experts were further asked to repeatedly test the developed models (see section “BBN validation and testing” for more details). The CPTs were finalized after these tests, so that the overall behavior aligned with the feedback of the experts.

### BBN validation and testing

The BBN for each group of stream types was trained and validated in GeNIe Modeler v.2.5 (BayesFusion, LLC, 2020) by applying threefold cross-validation. During threefold cross-validation, the dataset was randomly split into three subsets, or folds. The model was then trained on two folds, whereas one fold was hold out to test the model; the procedure was repeated three times. The resulting accuracies per node as well as for the whole model were averaged and represent the proportion of alignment between observed and predicted node states. For this validation procedure, the data sets for the groups of stream types were discretized according to the BBNs’ node states.

In addition, experts tested the BBNs’ diagnostic accuracy with their own monitoring data for single sites. Therefore, the BBNs were implemented as online tools using the shiny package in R (Chang et al., 2021). The default settings of these tools represent the joint probability distributions across states of metrics and potential degradation causes as represented in the data underlying the models’ development. Per group of stream types, the input mask allows the experts to choose the corresponding state per biological metric. Applying the experts’ specifications, the tools calculate probabilities of changes in potential stressors in relation to the default settings and order them from the strongest to the weakest change. This order reflects the putative hierarchy of potential degradation causes impacting the relevant site. The experts then compared the identification and hierarchy of stressors as provided by the tools with their knowledge about actual occurring stressors. Based on their expertise, they provided information on the reliability of the diagnosed stressors as ‘overestimated,’ ‘underestimated,’ ‘incorrectly diagnosed,’ or ‘correctly diagnosed’ (Figs. S1–S3). The proportion of ‘correctly diagnosed’ stressors constitutes the expert-based accuracy. The BBN for the group ‘streams of the low mountain ranges’ was tested for 31 monitoring sites, the BBN for the group ‘streams/rivers of the pre-alpine region’ for nine sites, and the BBN for the group ‘rivers of the low mountain ranges’ for 16 sites.

### Diagnostic value of information

GeNIe modeler v.2.5 (BayesFusion, LLC, 2020) supports diagnosis by calculating a diagnostic value expressed as ‘entropy reduction.’ The calculation is based on cross-entropy, an information–theoretical performance measure. In information theory, entropy quantifies the uncertainty associated with a possible event of a random variable (Cover & Thomas, 2006; Shannon, 1948). Accordingly, cross-entropy measures the expected reduction in entropy between two probability distributions of a possible event of a random variable (possible state of a potential degradation cause) given knowledge of observable variables (biological metrics). In this study, entropy reduction associated with a potential degradation cause was averaged over all its possible states. The more a biological metric reduces the entropy associated with a potential degradation cause, the higher this metric’s value regarding discriminatory diagnosis.

### Assessment metrics vs. diagnostic metrics

We tested if the accuracy of stressor diagnosis is affected by the use of diagnostic metrics, in addition to the assessment metrics. Theoretically, a diagnostic metric can be any of the more than 300 macroinvertebrate community metrics (e.g., share of taxa with habitat and zonation preferences or particular feeding types) that are calculated by the German assessment tool PERLODES (www.gewaesser-bewertung-berechnung.de). In contrast, an assessment metric (e.g., German Fauna Index, Saprobic Index) is a metric that has been identified to reliably indicate the ecological status of streams and rivers in line with the WFD (Meier et al., 2006b, compare header of Fig. 3). The stressor hierarchies and their assessments, resulting from indicating the states of as many metrics included in the respective BBN as possible, were provided by the experts (Figs. S1–S3). Additionally, the stressor hierarchies exclusively based on the assessment metrics were calculated. Both hierarchies were compared in terms of the probabilities for each individual stressor. If the change in probability for a stressor was lower than 10%, no change in diagnostic accuracy and stressor hierarchy was assumed. If the probability changed by more than 10%, it was checked whether the new stressor hierarchy got closer to the expert opinion or not. If yes, it was classified as ‘correctly diagnosed.’ A stressor was classified as under- or overestimated, if its probability changed by more than half of the probability that resulted from providing solely the states of assessment metrics.

## Results

### BBNs

Across the three BBNs, 13 potential stressors (Table 2) could be conceptually associated with 18 diagnostically useful metrics (Table 3).

**Table 2:** Overview of biological metrics included in the three developed BBNs. Per metric, a short description and its potential identification capability is provided

**Metric**	**Metric description**	**Potential indication**
Epirhithral preference	Epirhithral: oxygen-rich, cold, gravelly sole, turbulent, irregular flow (% individuals)	Modified hydromorphology, increased water temperature, land use
Metarhithral preference	Metarhithral: oxygen-rich, cold, gravelly sole, turbulent, irregular flow (% individuals)	Modified hydromorphology, increased water temperature, land use
Hyporhithral preference	Hyporhithral: oxygen-rich, water temperature rarely ≥ 14 °C, gravelly to sandy sole, increased plant growth (% individuals)	Modified hydromorphology, land use
Littoral preferences	Littoral: interface between land and water, photo-synthetically active radiation, low flow conditions (% individuals)	Modified hydromorphology, land use
Pelal preference	Pelal: unconsolidated fine sediments; grain size < 0.063 mm (% individuals)	Modified hydromorphology, land use
Psammal preference	Psammal: sand; grain size 0.063–2 mm (% individuals)	Modified hydromorphology, fine sediment load, land use
Shredders	Individuals that decompose organic matter ( e.g., fallen leaves) (%)	Modified hydromorphology
Active filter feeders	Individuals that feed on suspended organic matter in the water (%)	Modified hydromorphology, land use
EPT taxa	Ephemeroptera, Plecoptera, and Trichoptera taxa [%]	Modified hydromorphology, increased water temperature, land use
EPTCBO taxa	Number of Ephemeroptera, Plecoptera, Trichoptera, Coleoptera, Bivalvia, and Odonata taxa	Modified hydromorphology, fine sediment load, land use
Coleoptera	Coleoperta taxa (%)	Modified hydromorphology
Trichoptera	Trichoptera taxa (%)	Modified hydromorphology, land use
SPEAR_pesticides_	Measure of macroinvertebrate community change due to short-term pulsed exposure to insecticides, fungicides and other pesticides	Land use
German Fauna Index	Ratio between taxa indicating (semi-)natural waterbodies and degraded waterbodies (as EQR)	Modified hydromorphology, land use
Rheoindex	Ratio of rheophilic/rheobiontic taxa to limnobiontic/limnophilic taxa [based on frequency classes]	Modified hydromorphology, fine sediment load, land use
KLIWA Index_MZB_	Temperature tendency of a macroinvertebrate community based on its species temperature tolerances and preferences	Increased water temperature
German Saprobic Index	Measure of saprobic status based on macroinvertebrate composition	Modified hydromorphology, increased water temperature, fine sediment load, land use, nutrient load
Trophic Diatom Index	Measure of trophic status based on diatom composition	Nutrient load

**Table 3:** Overview of potential degradation causes included in the three developed BBNs. If a stressor is included in the BBN of a specific group of stream types, mean (min–max) is given

**Stressor**	**Spatial scale/definition of stressors**	**Group of stream types**		
		**SLMR**	**SRPAR**	**RLMR**
**Land use**				
Agriculture	% of catchment of monitoring site	18.6 (0.0–73.8)	29.4 (0.0–70.8)	19.4 (0.0–64.17)
Intensive grassland	% of catchment of monitoring site		26.6 (1.0–66.8)	
Special crops	% of catchment of monitoring site	1.6 (0.0–55.5)	0.9 (0.0–20.2)	
Urbanized areas	% of catchment of monitoring site	6.5 (0.0–39.6)	7.2 (0.9–20.9)	9.1 (2.0–62.9)
**Hydromorphological conditions**				
Backwater	Mean assessment result (scale: 1–7) along a 5-km stretch upstream of monitoring site			5.3 (3.0–7.0)
Width variance	Mean assessment result (scale: 1–7) along a 5-km stretch upstream of monitoring site	4.8 (1.0–7.0)		
Flow diversity & depth variance	Mean assessment result (scale: 1–7) along a 5-km stretch upstream of monitoring site	4.1 (1.0–7.0)	4.2 (1.0–6.6)	4.6 (1.2–7.0)
Substrate diversity	Mean assessment result (scale: 1–7) along a 5-km stretch upstream of monitoring site	3.9 (1.0–7.0)	3.9 (1.0–6.5)	4.4 (1.2–6.3)
Bank condition	Mean assessment result (scale: 1–7) along a 5-km stretch upstream of monitoring site	3.5 (1.0–6.5)		3.4 (1.2–6.7)
Bank vegetation cover	Mean assessment result (scale: 1–7) along a 5-km stretch upstream of monitoring site		3.6 (1.5–7.0)	
**Others**				
Fine sediments	% of fine sediments (Psammal, Pseudopsammal, Agryllal, and FPOM) at monitoring site	6.7 (0.0–70.0)	5.0 (0.0–95.0)	6.1 (0.0–100.0)
Water temperature	If a pre-defined value of central temperature tendency for a macroinvertebrate community based on its species temperature tolerances and preferences (KLIWA Index_MZB_) at a monitoring site is exceeded, the probability of thermal pollution strongly increases (see Tables S1–S3)	12.4 (3.6–25.0)^a^	14.1 (6.5–18.6)^a^	16.1 (9.5–26.7)^a^
Nutrients	If a pre-defined value of the Trophic Diatom Index at a monitoring site is exceeded, the probability of nutrient pollution strongly increases (Tables S1–S3)	2.6 (1.3–3.3)^b^	2.6 (1.6–3.1)^b^	2.7 (1.5–3.3)^b^

^a^Values of KLIWA Index_MZB_

^b^Values of Trophic Diatom Index

**Fig. 2:**
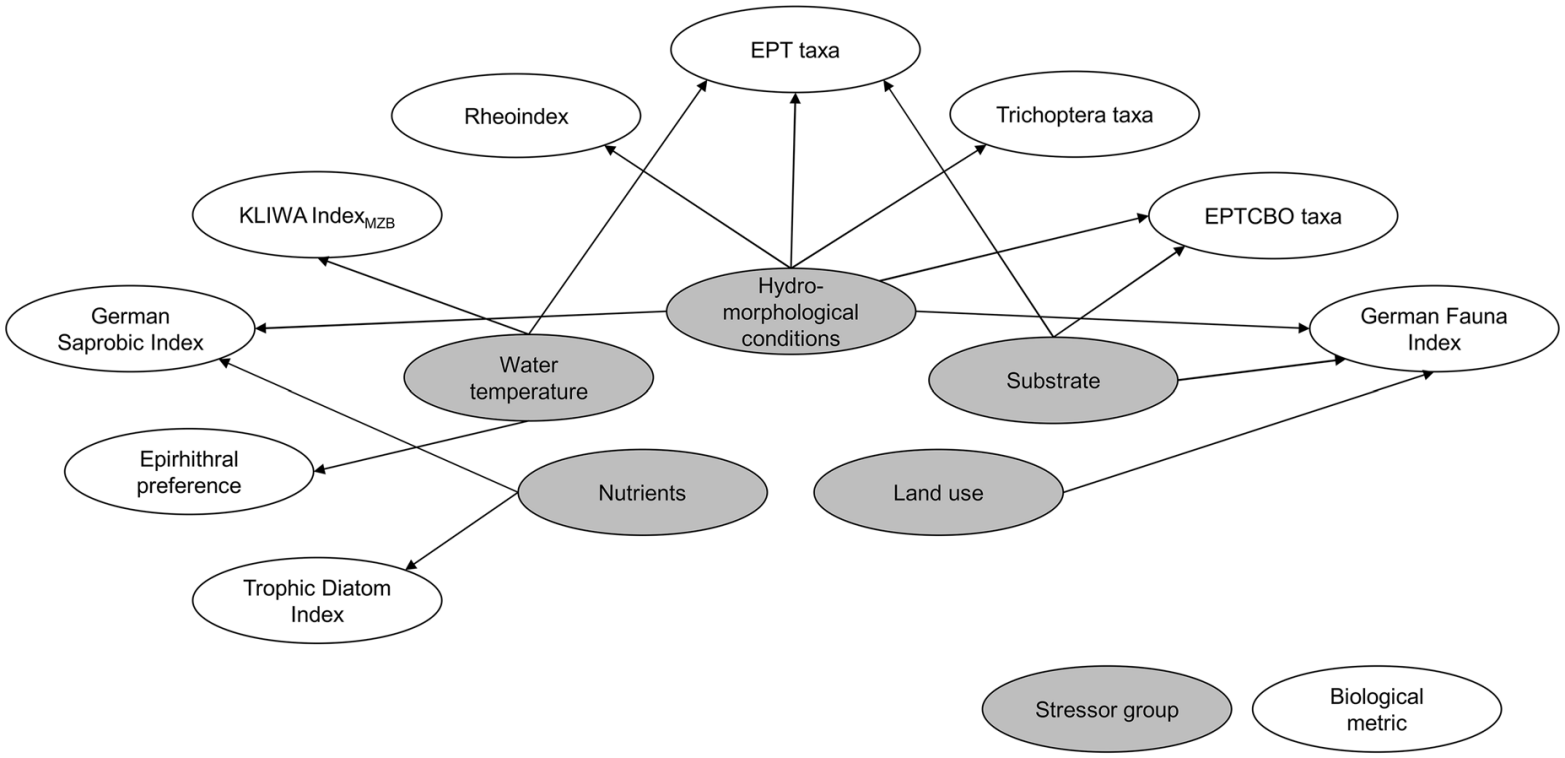
Shared diagnostic relationships between metrics and stressor groups across all groups of stream types

The BBNs for ‘streams of the low mountain ranges’ (Fig. 1C) and for ‘streams/rivers of the pre-alpine region’ (Fig. S4) both comprise 15 biological metrics and ten potential degradation causes. The BBN for ‘streams of the low mountain ranges’ (Fig. S5) includes 13 biological metrics and nine potential degradation causes. The stressor groups land use, substrate and hydromorphological conditions were included based on data, whereas the stressor groups nutrients and water temperature were included based on expert suggestions. Across the three BBNs, nine biological metrics were shown or assumed to diagnose one or more stressor groups (Fig. 2). The stressor group of hydromorphological conditions is linked to six of these metrics, whereas land use, for example, is only linked to one. Except for land use and nutrients, stressor groups are linked with metrics expressing taxonomic composition, biodiversity, and habitat preference.

**Fig. 3:**
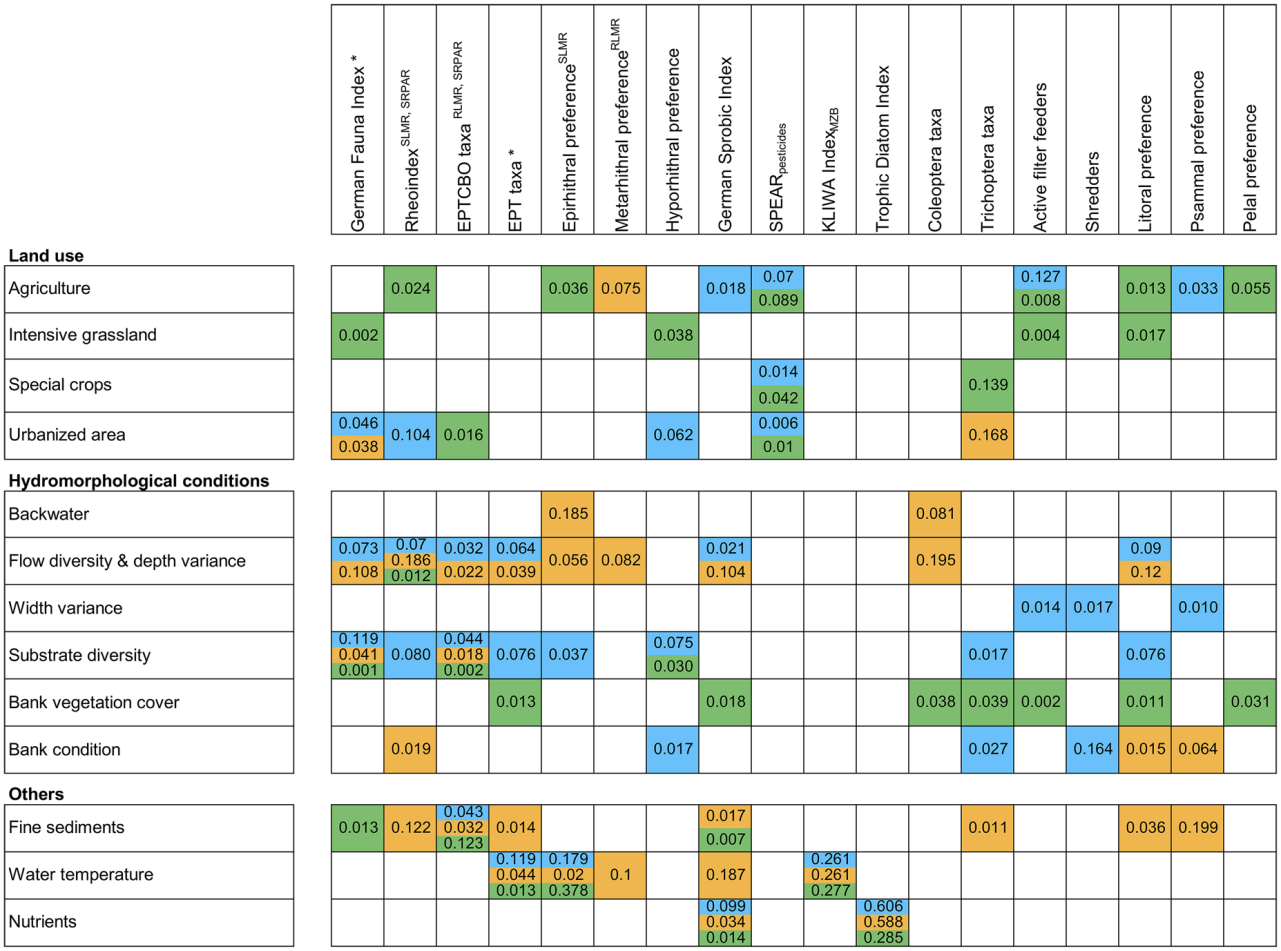
Numbers indicate the cross-entropy based diagnostic value of a metric to diagnose a potential degradation cause. The unit-less value ranges from 0 to 1; the higher the value, the better an observable variable (metric value) predicts a possible event (state of potential degradation cause). Relationships between biological metrics and degradation causes as displayed in the three developed BBNs. *Assessment metric for all groups of stream types, SLMR assessment metrics for ‘streams of the low mountain ranges,’ SRPAR assessment metrics for ‘streams/rivers of the pre-alpine region,’ RLMR assessment metrics for ‘rivers of the low mountain ranges’

The same sets of metrics are used for all three groups of stream types to diagnose flow diversity and depth variance, substrate diversity, fine sediments, water temperature, and nutrients (Fig. 3). These common (sets of) biological metrics are mainly related to taxonomic composition and biodiversity. For each group of stream types, these sets of ‘common metrics’ are complemented by additional metrics that are associated with the relevant degradation causes. The diagnostically useful metrics indicating bank conditions are not shared among groups of stream types; here, different metrics expressing habitat preferences are used for the three groups of stream types. Three biological metrics are only included in one of the BBNs: share of shredders (‘streams of the low mountain ranges’), share of taxa with pelal preference (‘streams/rivers of the pre-alpine region’), and share of taxa with metarhithral preference (‘rivers of the low mountain ranges’). Share of shredders contributes to discrimination of both width variance and bank condition, share of taxa with pelal preference to both agriculture and bank vegetation cover, and share of taxa with metarhithral preference to agriculture, flow diversity and depths variance, and water temperature, respectively.

Highest diagnostic values across all groups of stream types were calculated for the KLIWA Index_MZB_ and the Trophic Diatom Index (Fig. 3). Besides these, the highest diagnostic values per group of stream types were found for share of shredders (indicating bank condition for ‘streams of the low mountain ranges’), share of Trichoptera taxa (indicating the share of special crops in the catchment for ‘streams/rivers of the pre-alpine region’), as well as share of taxa with psammal preference (indicating fine sediments in ‘rivers of the low mountain ranges’).

### Model validation and testing

**Fig. 4:**
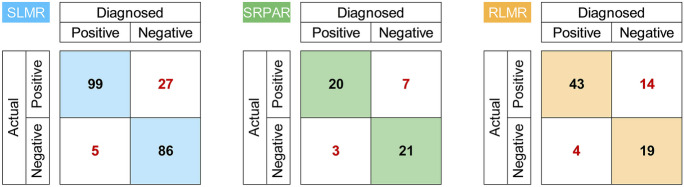
Expert evaluation results of the developed BBNs for ‘streams of the low mountain ranges’ (SLMR), ‘streams/rivers of the pre-alpine region’ (SRPAR), and ‘rivers of the low mountain ranges’ (RLMR). Positive indicates that the stressors were correctly diagnosed or observed in the field whereas negative indicates that the stressors were incorrectly identified or not observable in the field

**Fig. 5:**
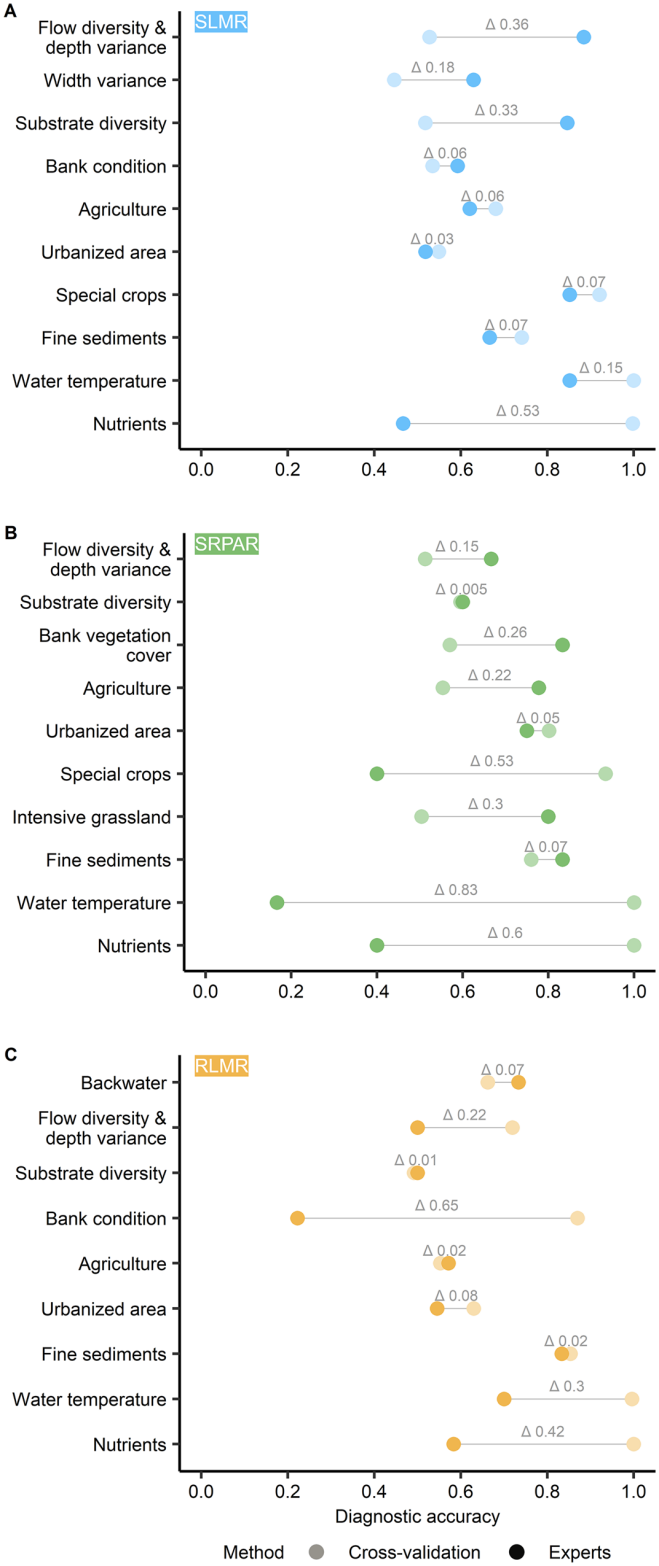
Comparison of diagnostic accuracy resulting from threefold cross-validation and expert opinion for the developed BBNs for ‘streams of the low mountain ranges’ (SLMR) (**A**), ‘streams/rivers of the pre-alpine region’ (SRPAR) (**B**), and ‘rivers of the low mountain ranges’ (RLMR) (**C**)

Based on expert judgment, the proportion of correctly diagnosed potential degradation causes clearly outweighs the incorrectly diagnosed ones for all three BBNs (Fig. 4). For ‘streams of the low mountain ranges,’ 85.5% were correctly identified, 80.4% for ‘streams/rivers of the pre-alpine region,’ and 77.5% for ‘rivers of the low mountain ranges.’ It needs to be noted, however, that these percentages do neither include diagnosed degradation causes the experts did not provide feedback on nor over- or underestimated degradation causes. Over- and underestimated degradation causes according to the experts’ evaluation, however, were considered for Fig. 5A–C. The BBNs’ tests by experts yielded poor to good diagnostic accuracies for potential degradation causes across the three groups of stream types, ranging from 17% for water temperature in the BBN for ‘streams/rivers of the pre-alpine region’ to 92% for flow diversity and depth variance in the BBN for ‘streams of the low mountain ranges.’ Cross-validation-based accuracies range between 44% for width variance in ‘streams of the low mountain ranges’ and 100% for water temperature and nutrients across all three groups of stream types.

With diagnostic accuracies of about 60% or higher, both in cross-validation and expert judgement, seven potential degradation causes were reliably diagnosed across the three BBNs. Only fine sediments were reliable diagnosed across all groups of stream types, whereas the remaining six potential degradation causes showed a more stream type group-specific outcome. Water temperature was reliably diagnosed for two groups of stream types (‘streams of the low mountain ranges’ and ‘rivers of the low mountain ranges’), while substrate diversity was reliably diagnosed only for ‘streams/rivers of the pre-alpine region.’ The diagnostic accuracies indicate a moderate performance of the developed BBNs. Expert-based accuracies are lower than those based on cross-validation in about half of the cases, even when cross-validation yielded accuracies > 60%. Thus, expert judgement seems more conservative in more than half of all cases compared to the results of cross-validation.

### Assessment metrics vs. diagnostic metrics

**Fig. 6:**
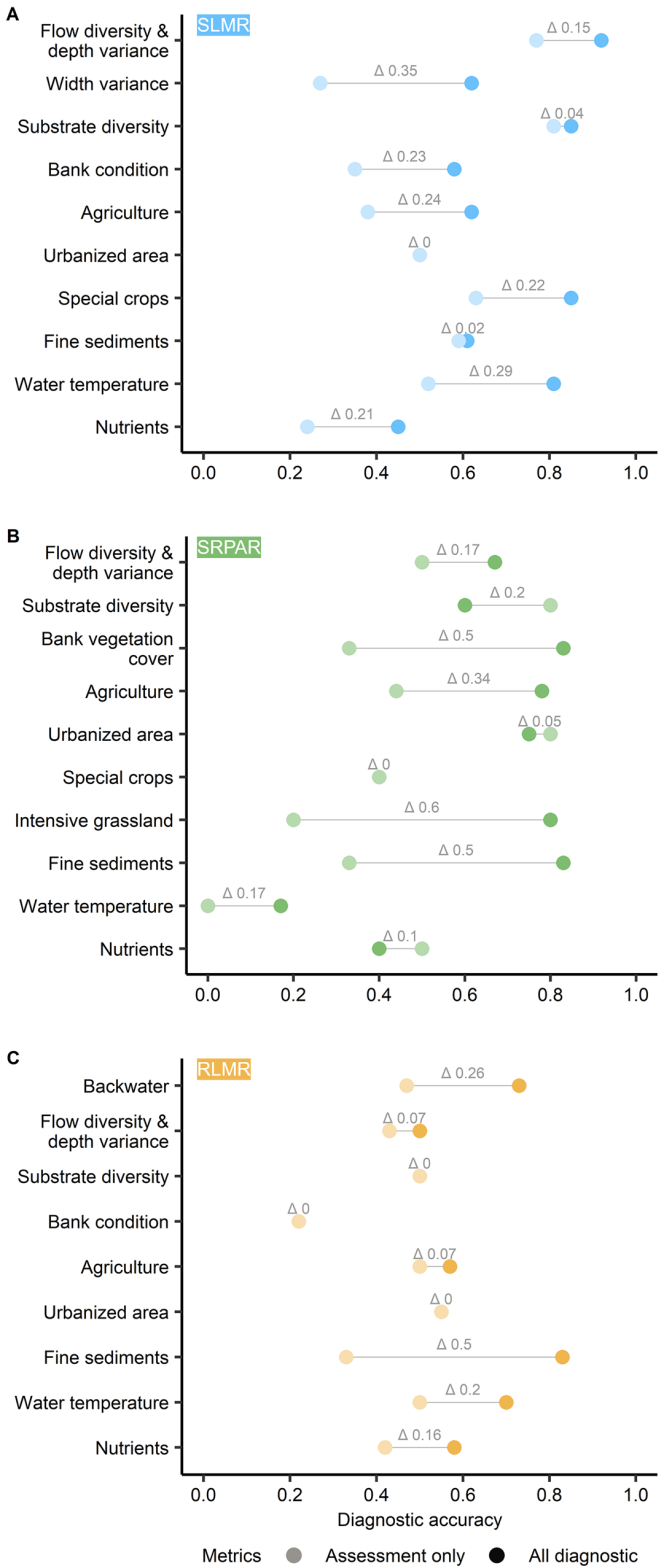
Comparison of diagnostic accuracy of the developed BBNs for ‘streams of the low mountain ranges’ (SLMR) (**A**), ‘streams/rivers of the pre-alpine region’ (SRPAR) (**B**), and ‘rivers of the low mountain ranges’ (RLMR) (**C**), if only river assessment metrics or all diagnostic metrics are considered

Across all three groups of stream types, the diagnostic accuracies of most potential degradation causes increase when values were not only provided for the assessment metrics but for all 12 or 15 diagnostic metrics (Fig. 6A–C). These increases range between 1 and 60%. The diagnostic accuracies for substrate diversity, bank condition, and urban areas for ‘streams of the low mountain ranges’ do not change. This also holds true for the diagnostic accuracy of urban areas for ‘streams of the low mountain ranges’ and for special crops for ‘streams/rivers of the pre-alpine region.’ Diagnostic accuracies for urbanized areas, substrate diversity, and nutrients in the BBN for ‘streams/ rivers of the pre-alpine region’ decrease when all diagnostic metrics are included.

## Discussion

### Diagnostic accuracy

The overall diagnostic accuracy of our BBNs demonstrates the general utility of biological metrics to reflect particular environmental states or conditions. Based on the experts’ evaluation, the three BBNs correctly identified, on average, 80% of the diagnosed potential degradation causes. For individual causes, up to 92% and 100% correct identifications were achieved by expert-based testing and data-based cross-validation, respectively. Irrespective of the stream types, the expert-based accuracies were the highest for hydromorphological causes including fine sediment load. In contrast, the data-based cross-validation identified the highest accuracies for warming and nutrient enrichment, two water quality-related causes. Further, the highest diagnostic values were calculated for the KLIWA Index_MZB_ and the Trophic Diatom Index. These findings seem contradictory but may be explained by shortcomings of the models’ structure and parameterization. Both biological indices were integrated to improve the approximation of corresponding stressor intensity, i.e., the probability of warming and excess nutrients were considered higher, if a previously defined threshold value (see Tables S1–S3 for stream type group-specific threshold values) was exceeded for the metrics. Hence, these strong stressor–metric relationships are owed to the models’ structure and parameterization, with only two states (cause present or absent) being implemented in the corresponding conditional probability tables. Besides these high diagnostic accuracies for individual degradation causes, other causes revealed weaker results. The diagnostic accuracy for bank modification in ‘streams of the low mountain ranges’ or substrate diversity in ‘rivers of the low mountain ranges,’ for example, were notably lower (50–58%). It is assumed that this was due to weak associations between structural modifications and biological metrics in our data.

The comparison of diagnostic and assessment metrics revealed that by a combination of both it was possible to achieve a higher diagnostic accuracy as compared to a model purely based upon assessment metrics. Across all models, eleven out of the 15 highest diagnostic values were associated with diagnostic metrics, i.e., metrics that are not regularly used for the ecological status assessment. The integration of such diagnostic metrics increased the diagnostic accuracy for the majority of potential degradation causes by up to 60%. The exceptions from this improvement were, for example, causes that were solely linked to assessment metrics, such as substrate diversity for ‘rivers of the low mountain ranges.’ Furthermore, the findings suggest that the discrimination between multiple causes of degradation requires multiple biological symptoms (metrics). We assume that the number of symptoms (metrics) used to diagnose multiple causes needs to be higher than the number of actual causes to discriminate.

### Discrimination between causes

Our findings align with those of previous studies (e.g., Clews & Ormerod, 2009; Mondy & Usseglio-Polatera, 2013; Statzner & Bêche, 2010) that observed the discrimination between individual causes (stressors) to be enabled by the simultaneous consideration of numerous biological metrics. This is notable, since the number of biological metrics that are linked to only one stressor (*N* = 7) is much lower than the number of metrics that are linked to more than one cause (*N* = 36) in the three BBNs. Both, community composition metrics (e.g., share of EPT taxa) and species trait metrics (e.g., proportion of feeding types) apparently respond to various sources of degradation (e.g., Gieswein et al., 2017; Hering et al., 2006; Pilière et al., 2016; Poff et al., 2006). Here, the highest diagnostic values per stressor–metric relationship were equally spread across the different metrics types.

The discrimination between the causes related to flow velocity and depth variation, bank condition, and fine sediment entry in the ‘rivers of the low mountain ranges,’ for example, would be impossible, if all these causes were linked solely to the Rheoindex. The Rheoindex presents the relationship between rheophilic/rheobiont taxa and limnophilic/limnobiont taxa (Banning, 1998). Hence, the index is not only directly related to flow diversity (and its modification) but also indirectly related to its concomitants bottom substrate modifications and changes in the nutrient concentrations (Meier et al., 2006b). The discrimination between all these potential causes improved, if additional metrics were integrated. Macroinvertebrate taxa associated with the metarhithral are also adapted to higher flow velocities, but usually prefer a water temperature below 18 °C (Moog & Wimmer, 1994). This is why rheophilic taxa occur in fast-flowing streams and larger rivers, while metarhithral-preferring taxa are largely limited to the metarhithral, i.e., mid-sized streams (Meier et al, 2006b). Taxa with psammal preference are adapted to sandy bottom substrates. They could be linked to effects of increased fine sediment entry and hence support the discrimination between flow modification and fine sediment entry. The combination of the Rheoindex, and the share of taxa with metarhithral and psammal preferences in a BBN hence can help to better discriminate between the potential causes flow modification, substrate modification and warming effects.

Further analyses with a larger dataset might reveal stronger and diagnostically more useful relationships. This assumption is supported by the finding that macroinvertebrate metrics turned out to respond more sensitive to land use and hydromorphological causes than to those causes related to water quality. This aligns with the findings of Hering et al. (2006) and points at the need to integrate other organism groups (e.g., diatoms, fish, and aquatic macrophytes) in the diagnosis. We believe that a multi-organismal diagnosis could improve the discrimination between multiple causes and particularly contribute to a better integration of causes that act at different spatial scales. Fish are known to respond to longitudinal river characteristics over several kilometers or even tens of kilometers (Bunn & Arthington, 2002; Gido et al., 2015; Poff, 1997). Therefore, incorporating fish diagnostic metrics may increase the diagnostic accuracy of alterations of longitudinal river characteristics, such as dams, stagnant conditions, and enhanced water temperature. The aquatic flora, including diatoms and macrophytes, is physiologically directly related to nutrient concentrations (Poikane et al., 2018; Schneider & Melzer, 2003), flow velocity, and substrate coarseness (Kaijser et al., 2022) and are thus presumably well suited to diagnose related causes of degradation.

### Benefits and potential application of the diagnostic tools

Three features of BBNs turned out to be particularly advantageous during the development process: (1) the graphical representation of stressor-metric relationships, (2) the possibility to combine different sources of evidence, and (3) the inherent capability to deal with uncertainty. The graphical representation facilitated the exchange with experts during the workshops. In contrast to purely data-based approaches currently used for stressor diagnosis in aquatic systems, our approach is not limited by data availability. For example, Dézerald et al. (2020) had to exclude a pressure category from their diagnosis due to insufficient data, whereas our approach allows such data limitations to be overcome with other types of evidence, such as expert knowledge. Examples from our study include the implementation of the potential degradation causes water temperature and nutrients. Alike other diagnostic tools such as CADDIS (U.S. EPA, 2017) and Eco Evidence (Nichols et al., 2011), our approach also allows to analyze the strength of evidence and thus the quantification of uncertainties. In our approach, this is achieved by a definition of conditional probabilities. The more the states of an effect variable can be distinguished by probability distributions in the underlying CPTs, the lower the uncertainty associated with the cause-and-effect relationship in the diagnostic BBN. For example, if there is strong evidence that a high KLIWA Index_MZB_ is associated with thermal pollution (warming), the conditional probability for a high metric value under warming condition would be set to 80% or even higher. This corresponds to an uncertainty of 20%, i.e., in 20% of the cases a high metric value might be found at sites without warming conditions. Hence, the principle of assigning conditional probabilities directly allows to integrate and quantify uncertainties in BBN models.

Even though the diagnostic accuracy of some degradation causes showed modest results, the developed BBNs are applicable for their main purposes (Feld et al., 2020): informing decisions by estimating changes in probabilities of potential degradation causes and ranking them hierarchically. At this point, it should be emphasized that the developed tools are not intended to replace existing tools for ecological status assessment according to the WFD or the invaluable knowledge of numerous domain experts in river authorities and spatial planning offices. Rather, our diagnostic tools are meant to complement them. When the potential causes of degradation are just unknown, probabilistic diagnostic tools can make a decisive contribution to both the identification of actual stressors and the exclusion of improbable stressors, especially at the beginning of the diagnostic process.

## Conclusions

Ecological status assessment according to the WFD is often based on a few community-based assessment metrics. While a few assessment metrics allow to integrate the impacts of multiple potential causes of degradation, these metrics do not allow to trace back from an assessment result to individual potential causes of degradation. Diagnostic metrics can help to reverse the integration and discriminate between different stressors. The alignment of metrics and stressors through cause-and-effect relationships provides the foundation of BBNs. Even though the diagnostic accuracy of single degradation causes can be further improved, our findings confirm the discriminatory power of three diagnostic BBNs that were built upon regular monitoring data and experts’ knowledge on streams and rivers in the Federal State of Baden-Württemberg (Germany). Validation and testing of the diagnostic BBNs confirmed their usefulness regarding both the identification of causes of degradation and their hierarchical classification. If embedded in web-based applications, the BBNs can be easily applied by a wide range of end users to support the identification of causes of deteriorated ecological status of streams and rivers. Hence, such web-based diagnostic tools can help river basin managers better link the outcome of ecological status assessments with related programs of management measures. The diagnostic tools on the three BBNs presented in this study are available at https://www.lubw.baden-wuerttemberg.de/wasser/diagnosetool-makrozoobenthos.

### Supplementary Information

Below is the link to the electronic supplementary material.

Supplementary file1 (PDF 1.45 MB)

## Data Availability

The data that support the findings of this study are available from the State Agency for the Environment of Baden-Württemberg (Germany), but restrictions apply to the availability of these data, which were used under license for the current study, and so are not publicly available. Data are however available upon reasonable request from the State Agency for the Environment of Baden-Württemberg (Germany). A functional English version of the diagnostic tool has been made available on GitHub: https://github.com/katharinarettig/diagnostic_tools.
