# Supplementary material for: Of causes and symptoms: using monitoring data and expert knowledge to diagnose the causes of stream degradation
Source: Environ Monit Assess. 2023 Sep 28;195(10):1253. doi: 10.1007/s10661-023-11741-5 (PMC10539194; doi:10.1007/s10661-023-11741-5)
Supplement: Supplementary file 1 — Supplementary file1 (PDF 1.45 MB) [file 10661_2023_11741_MOESM1_ESM.pdf]

Supplementary material

Of causes and symptoms: using monitoring data and expert knowledge to diagnose causes of stream degradation

Katharina Rettig[1,*], Renate Semmler-Elpers[2], Denise Brettschneider[2], Daniel Hering[1,3], Christian K. Feld[1,3]

[1] Faculty of Biology, Aquatic Ecology, University of Duisburg-Essen, Universitätsstr. 5, Essen, 45141, Germany
[2] State Agency for the Environment Baden-Württemberg, Griesbachstr. 1, Karlsruhe, 76185, Germany
[3] Center for Water and Environmental Research, University of Duisburg-Essen, Universitätsstr. 2, Essen, 45141, Germany

* Corresponding author: katharina.rettig@uni-due.de

Table S1 Overview of nodes and their states of the BBN for 'streams of the low mountain ranges'.

	Variable	States	Ranges
**Potential degradation causes**	Agriculture<br>% of catchment of monitoring site	Low<br>Medium<br>High	≤ 10<br>> 10 - ≤ 20<br>> 20
	Special crops<br>% of catchment of monitoring site	Low<br>High	≤ 5<br>> 5
	Urbanized area<br>% of catchment of monitoring site	Low<br>Medium<br>High	≤ 5<br>> 5 - ≤ 10<br>> 10
	Flow velocity & depth variance<br>Mean assessment result (scale: 1-7) along a 5 km stretch upstream of monitoring site	Natural to moderately modified<br>Significantly to heavily modified<br>Very heavily to completely modified	≤ 3<br>> 3 - ≤ 5<br>> 5
	Width variance<br>Mean assessment result (scale: 1-7) along a 5 km stretch upstream of monitoring site	Natural to significantly modified<br>Heavily to very heavily modified<br>Completely modified	≤ 4<br>> 4 - ≤ 6<br>> 6
	Substrate diversity<br>Mean assessment result (scale: 1-7) along a 5 km stretch upstream of monitoring site	Natural to moderately modified<br>Significantly to heavily modified<br>Very heavily to completely modified	≤ 3<br>> 3 - ≤ 4.5<br>> 4.5
	Bank condition<br>Mean assessment result (scale: 1-7) along a 5 km stretch upstream of monitoring site	Natural to significantly modified<br>Heavily to completely modified	≤ 4<br>> 4
	Fine sediments<br>% of fine sediments (Psammal, Pseudopsammal, Agryllal, and FPOM) at monitoring site	Low<br>Medium<br>High	≤ 10<br>> 10 - ≤ 20<br>> 20
	Water temperature	No<br>Yes	High probability if KLIWA-Index$_{MZB}$ is 'Low'<br>High probability if KLIWA-Index$_{MZB}$ is 'High'
	Nutrients	No<br>Yes	High probability if Trophic Diatom Index is 'Low' or 'Medium'<br>High probability if Trophic Diatom Index is 'High'

Table S1 continued

	Variable	States	Ranges
Biological metrics	German Fauna Index<br>as Environmental Quality Ratio (EQR)	Low<br>Medium<br>High	≤ 0.4<br>> 0.4 - ≤ 0.6<br>> 0.6
	Rheoindex	Low<br>Medium<br>High	≤ 0.75<br>> 0.75 - ≤ 0.85<br>> 0.85
	EPTCBO taxa<br>number of taxa	Low<br>Medium<br>High	≤ 20<br>> 20 - ≤ 30<br>> 30
	EPT taxa<br>% taxa	Low<br>Medium<br>High	≤ 40<br>> 40 - ≤ 50<br>> 50
	Epirhithral preference<br>% individuals	Low<br>Medium<br>High	≤ 13<br>> 13 - ≤ 17<br>> 17
	Hyporhithral preference<br>% individuals	Low<br>Medium<br>High	≤ 15<br>> 15 - ≤ 20<br>> 20
	German Saprobic Index	Low<br>Medium<br>High	≤ 1.5<br>> 1.5 - ≤ 2.0<br>> 2.0
	$SPEAR_{pesticides}$	Low<br>Medium<br>High	≤ 22<br>> 22 - ≤ 33<br>> 33
	KLIWA $Index_{MZB}$<br>central temperature tendency for a macroinvertebrate community (°C)	Low<br>High	≤ 15<br>> 15
	Trophic Diatom Index	Low<br>Medium<br>High	≤ 2.7<br>> 2.7 - ≤ 3.15<br>> 3.15

Table S1 continued

	Variable	States	Ranges
**Biological metrics**	Trichoptera taxa<br>% taxa	Low<br>Medium<br>High	≤ 10<br>> 10 - ≤ 20<br>> 20
	Active filter feeders<br>% individuals	Low<br>High	≤ 2.5<br>> 2.5
	Shredders<br>% individuals	Low<br>Medium<br>High	≤ 10<br>> 10 - ≤ 20<br>> 20
	Littoral preference<br>% individuals	Low<br>Medium<br>High	≤ 5<br>> 5 - ≤ 10<br>> 10
	Psammal preference<br>% individuals	Low<br>High	≤ 5<br>> 5

Table S2 Overview of nodes and their states of the BBN for 'streams / rivers of the pre-alpine region'.

	Variable	States	Ranges
**Potential degradation causes**	Agriculture<br>% of catchment of monitoring site	Low<br>Medium<br>High	≤ 20<br>> 20 - ≤ 40<br>> 40
	Intensive grassland<br>% of catchment of monitoring site	Low<br>Medium<br>High	≤ 20<br>> 20 - ≤ 40<br>> 40
	Special crops<br>% of catchment of monitoring site	Low<br>High	≤ 5<br>> 5
	Urbanized area<br>% of catchment of monitoring site	Low<br>High	≤ 10<br>> 10
	Flow velocity & depth variance<br>Mean assessment result (scale: 1-7) along a 5 km stretch upstream of monitoring site	Natural to significantly modified<br>Heavily to completely modified	≤ 4.5<br>> 4.5
	Substrate diversity<br>Mean assessment result (scale: 1-7) along a 5 km stretch upstream of monitoring site	Natural to significantly modified<br>Heavily to completely modified	≤ 4<br>> 4
	Bank vegetation cover<br>Mean assessment result (scale: 1-7) along a 5 km stretch upstream of monitoring site	Natural to moderately modified<br>Significantly to heavily modified<br>Very heavily to completely modified	≤ 3<br>> 3 - ≤ 5<br>> 5
	Fine sediments<br>% of fine sediments (Psammal, Pseudopsammal, Agryllal, and FPOM) at monitoring site	Low<br>Medium<br>High<br>Very High	≤ 10<br>> 10 - ≤ 20<br>> 20 - ≤ 40<br>> 40
	Water temperature	No<br>Yes	High probability if KLIWA-Index$_{MZB}$ is 'Low'<br>High probability if KLIWA-Index$_{MZB}$ is 'High'
	Nutrients	No<br><br>Yes	High probability if Trophic Diatom Index is 'Low' or 'Medium'<br>High probability if Trophic Diatom Index is 'High'

Table S2 continued

	Variable	States	Ranges
**Biological metrics**	German Fauna Index<br>as Environmental Quality Ratio (EQR)	Low<br>Medium<br>High	≤ 0.4<br>> 0.4 - ≤ 0.6<br>> 0.6
	Rheoindex	Low<br>Medium<br>High	≤ 0.6<br>> 0.6 - ≤ 0.75<br>> 0.75
	EPTCBO taxa<br>number of taxa	Low<br>Medium<br>High	≤ 20<br>> 20 - ≤ 30<br>> 30
	EPT taxa<br>% taxa	Low<br>Medium<br>High	≤ 35<br>> 35 - ≤ 50<br>> 50
	Epirhithral preference<br>% individuals	Low<br>Medium<br>High	≤ 10<br>> 10 - ≤ 15<br>> 15
	Hyporhithral preference<br>% individuals	Low<br>Medium<br>High	≤ 20<br>> 20 - ≤ 25<br>> 25
	German Saprobic Index	Low<br>Medium<br>High	≤ 1.7<br>> 1.7 - ≤ 2.0<br>> 2.0
	$SPEAR_{pesticides}$	Low<br>Medium<br>High	≤ 22<br>> 22 - ≤ 33<br>> 33
	KLIWA $Index_{MZB}$<br>central temperature tendency for a ma-croinvertebrate community (°C)	Low<br>High	≤ 14<br>> 14
	Trophic Diatom Index	Low<br>Medium<br>High	≤ 2.7<br>> 2.7 - ≤ 3.15<br>> 3.15

Table S2 continued

	Variable	States	Ranges
**Biological metrics**	Trichoptera taxa<br>% taxa	Low<br>Medium<br>High	≤ 5<br>> 5 - ≤ 10<br>> 10
	Coleoptera taxa<br>% taxa	Low<br>High	≤ 10<br>> 10
	Active filter feeders<br>% individuals	Low<br>High	≤ 2.5<br>> 2.5
	Littoral preference<br>% individuals	Low<br>Medium<br>High	≤ 5<br>> 5 - ≤ 10<br>> 10
	Pelal preference<br>% individuals	Low<br>High	≤ 10<br>> 10

Table S3 Overview of nodes and their states of the BBN for 'rivers of the low mountain ranges'.

	Variable	States	Ranges
Potential degradation causes	Agriculture<br>% of catchment of monitoring site	Low<br>Medium<br>High	≤ 10<br>> 10 - ≤ 30<br>> 30
	Urbanized area<br>% of catchment of monitoring site	Low<br>High	≤ 10<br>> 10
	Backwater<br>Mean assessment result (scale: 1-7) along a 5 km stretch upstream of monitoring site	Natural to significantly modified<br>Heavily to completely modified	≤ 5<br>> 5
	Flow diversity & depth variance<br>Mean assessment result (scale: 1-7) along a 5 km stretch upstream of monitoring site	Natural to significantly modified<br>Heavily to completely modified	≤ 4.5<br>> 4.5
	Substrate diversity<br>Mean assessment result (scale: 1-7) along a 5 km stretch upstream of monitoring site	Natural to significantly modified<br>Heavily modified<br>Very heavily to completely modified	≤ 4<br>> 4- ≤ 5<br>> 5
	Bank condition<br>Mean assessment result (scale: 1-7) along a 5 km stretch upstream of monitoring site	Natural to moderately modified<br>Significantly to completely modified	≤ 3<br>> 3
	Fine sediments<br>% of fine sediments (Psammal, Pseudopsammal, Agryllal, and FPOM) at monitoring site	Low<br>High	≤ 10<br>> 10
	Water temperature	No<br>Yes	High probability if KLIWA-Index$_{MZB}$ is 'Low'<br>High probability if KLIWA-Index$_{MZB}$ is 'High'
	Nutrients	No<br><br>Yes	High probability if Trophic Diatom Index is 'Low' or 'Medium'<br>High probability if Trophic Diatom Index is 'High'
Biological metics	German Fauna Index<br>as Environmental Quality Ratio (EQR)	Low<br>Medium<br>High	≤ 0.4<br>> 0.4 - ≤ 0.6<br>> 0.6
	Rheoindex	Low<br>Medium<br>High	≤ 0.6<br>> 0.6 - ≤ 0.8<br>> 0.8
	EPTCBO taxa<br>number of taxa	Low<br>Medium<br>High	≤ 15<br>> 15 - ≤ 25<br>> 25

Table S3 continued

	Variable	States	Ranges
**Biological metics**	EPT taxa<br>% taxa	Low<br>Medium<br>High	≤ 40<br>> 40 - ≤ 50<br>> 50
	Epirhithral preference<br>% individuals	Low<br>Medium<br>High	≤ 12<br>> 12 - ≤ 17<br>> 17
	Metarhithral preference<br>% individuals	Low<br>Medium<br>High	≤ 15<br>> 15 - ≤ 25<br>> 25
	German Saprobic Index	Low<br>Medium<br>High	≤ 1.7<br>> 1.7 - ≤ 2.1<br>> 2.1
	KLIWA Index$_{MZB}$<br>central temperature tendency for a macroinvertebrate community (°C)	Low<br>High	≤ 14<br>> 14
	Trophic Diatom Index	Low<br>Medium<br>High	≤ 2.7<br>> 2.7 - ≤ 3.15<br>> 3.15
	Trichoptera taxa<br>% taxa	Low<br>Medium<br>High	≤ 10<br>> 10 - ≤ 20<br>> 20
	Coleoptera taxa<br>% taxa	Low<br>Medium<br>High	≤ 5<br>> 5 - ≤ 10<br>> 10
	Littoral preference<br>% individuals	Low<br>Medium<br>High	≤ 5<br>> 5 - ≤ 10<br>> 10
	Psammal preference<br>% individuals	Low<br>High	≤ 7.5<br>> 7.5

Table S4 Overview of experts involved in the development process of the Bayesian Belief Networks and their validation.

Expert	Affiliation	Workshop 1	Test 1	Workshop 2	Test 2
1	District Administration Esslingen	x	x	x	x
2	Limnocon	x		x	
3	State Agency for the Environment Baden-Württemberg	x		x	
4	District Administration Tübingen	x	x	x	x
5	HBio-Hessen	x			
6	State Agency for the Environment Baden-Württemberg	x		x	
7	Infrastruktur & Umwelt	x	x	x	x
8	District Administration Tübingen	x			
9	District Administration Freiburg	x	x	x	x
10	Spang.Fischer.Natschka	x		x	
11	District Administration Karlsruhe	x	x	x	x
12	Ministry of the Environment, Climate Protection and the Energy Sector Baden-Württemberg	x		x	
13	Büro am Fluss	x			
14	State Agency for the Environment Baden-Württemberg	x	x	x	x
15	State Agency for the Environment Baden-Württemberg			x	
16	ALAND		x	x	
17	Infrastruktur & Umwelt			x	
18	District Administration Stuttgart			x	
19	Büro am Fluss			x	
20	District Administration Freiburg			x	
21	Büro am Fluss			x	
22	District Administration Karlsruhe		x	x	x

Monitoring site (SLMR_1 – SLMR_16)

		SLMR_1	SLMR_2	SLMR_3	SLMR_4	SLMR_5	SLMR_6	SLMR_7	SLMR_8	SLMR_9	SLMR_10	SLMR_11	SLMR_12	SLMR_13	SLMR_14	SLMR_15	SLMR_16
Hydromorpho-logical conditions	Flow diversity & depth variance		=		=			=		=	=	=	=	=	=	=	=
	Width variance		=	=	=			-			↓	=	=	↓	=	↓	=
	Substrate diversity		=		=			=		↑	=	=	=	=	=	=	=
	Bank condition		=	+	=			-			=	=	=	-	=	=	=
Land use	Agriculture		=	=	+		=	↓	=	↓	=	=	=	=	=	=	=
	Urbanized area		↑	=	=	=	=	↑			-	=	=	↓	=	-	-
	Special crops	=	=	=	+			=			=	=	=	↓	=	-	=
Others	Fine sediments		=		=	=		=	=	↑		=	=	=	=	=	=
	Water temperature		=	=	=			=	=			=	=	=	=	=	=
	Nutrients	-	-	=	=	↓		↓	-		=	=	=	=	=	=	-

Monitoring site (SLMR_17 – SLMR_31)

		SLMR_17	SLMR_18	SLMR_19	SLMR_20	SLMR_21	SLMR_22	SLMR_23	SLMR_24	SLMR_25	SLMR_26	SLMR_27	SLMR_28	SLMR_29	SLMR_30	SLMR_31
Hydromorpho-logical conditions	Flow diversity & depth variance	=	=	=	=	=	↓	=	=	=	=	=	=	=	↑	=
	Width variance	=	=	-	-	-	=	=	↓	↓	=	=	=	=	↑	=
	Substrate diversity	=	=	=	=	=	=	=	↑	=	=	=	=	=	↑	-
	Bank condition	=	=	-	↓	=	=	=	-	-	↓	-	-	-	=	=
Land use	Agriculture	↑	-	=	=	↑	=	=	↑	↑	=	↓	=	↓	↑	=
	Urbanized area	↑	=	↓	=	-	=	=	-	↓	↓	=	=	=	↓	+
	Special crops	=	=	=	=	=	=	=	=	=	=	=	=	=	+	=
Others	Fine sediments	=	=	=	↑	=	↑	=	↑	↑	↑	↑	↑	↑	↑	=
	Water temperature	↑	=	=	↑	=	↑	=	=	=	=	=	=	=	↑	=
	Nutrients	↓	=	-	=	=	↓	=	↓	↓	↓	↓	=	-	-	↓

\+ Stressor diagnosed but not actually present; - Stressor not diagnosed but actually present; ↑ Correctly diagnosed but hierarchically overestimated; ↓ Correctly diagnosed but hierarchically underestimated; = Correctly diagnosed; (blank) Unknown

Fig. S1 Expert knowledge-based reliability of the BBN for 'streams of the low mountain ranges'. For each monitoring site, diagnosed stressors and their hierarchy were compared with actually present stressors by the domain experts.

		Monitoring site								
		SRPAR_1	SRPAR_2	SRPAR_3	SRPAR_4	SRPAR_5	SRPAR_6	SRPAR_7	SRPAR_8	SRPAR_9
Hydromorpho-logical conditions	Flow diversity & depth variance	-		↓			=	=	=	=
	Bank vegetation cover	=			=		=	=	=	=
	Substrate diversity	-					=	=	=	-
Land use	Agriculture	=	=	=	=	=	=	=	-	=
	Urbanized area	=					=	=	=	-
	Special crops				+		=	=	+	↓
	Intensive grassland	-					=	=	=	=
Others	Fine sediments		=	↑	=	=			=	=
	Water temperature	↑	↑	↑				↑	=	+
	Nutrients	=				↓	=	=	=	-

\+ Stressor diagnosed but not actually present; - Stressor not diagnosed but actually present; ↑ Correctly diagnosed but hierarchically overestimated; ↓ Correctly diagnosed but hierarchically underestimated; = Correctly diagnosed; (blank) Unknown

Fig. S2 Expert knowledge-based reliability of the BBN for ‘streams / rivers of the pre-alpine region’. For each monitoring site, diagnosed stressors and their hierarchy were compared with actually present stressors by the domain experts. ‘Unknown’ if no evaluation was provided by the experts.

		Monitoring site															
		RLMR_1	RLMR_2	RLMR_3	RLMR_4	RLMR_5	RLMR_6	RLMR_7	RLMR_8	RLMR_9	RLMR_10	RLMR_11	RLMR_12	RLMR_13	RLMR_14	RLMR_15	RLMR_16
**Hydromorpho-logical conditions**	Backwater	+	=	=	=	=	=		=	+	↑	=	=	=	=	↑	=
	Flow diversity & depth variance		=	-	-	=		+	=	↑	=	↑	=	=	↑	↑	=
	Bank condition			↓	-				=		↑		-	-	↓	↓	=
	Substrate diversity			-	-				=				=	=	↓	↑	=
**Land use**	Agriculture			=	=	↓	=	↓	=	↑	=	↑	=	=	↓	↑	=
	Urbanized area			-	=		=		=	↓	=		-	↑	↑	=	=
**Others**	Fine sediments			=		=	=	+	=	=		=	=	=	=	↑	=
	Water temperature			-	=	=	↑		=			=		=	↑	=	=
	Nutrients		-	↓	=				=	-	=	-	=	=	=	-	=

\+ Stressor diagnosed but not actually present

\- Stressor not diagnosed but actually present

↑ Correctly diagnosed but hierarchically overestimated

↓ Correctly diagnosed but hierarchically underestimated

= Correctly diagnosed

(blank) Unknown

Fig. S3 Expert knowledge-based reliability of the BBN for ‘rivers of the low mountain ranges’. For each monitoring site, diagnosed stressors and their hierarchy were compared with actually present stressors by the domain experts. ‘Unknown’ if no evaluation was provided by the experts.

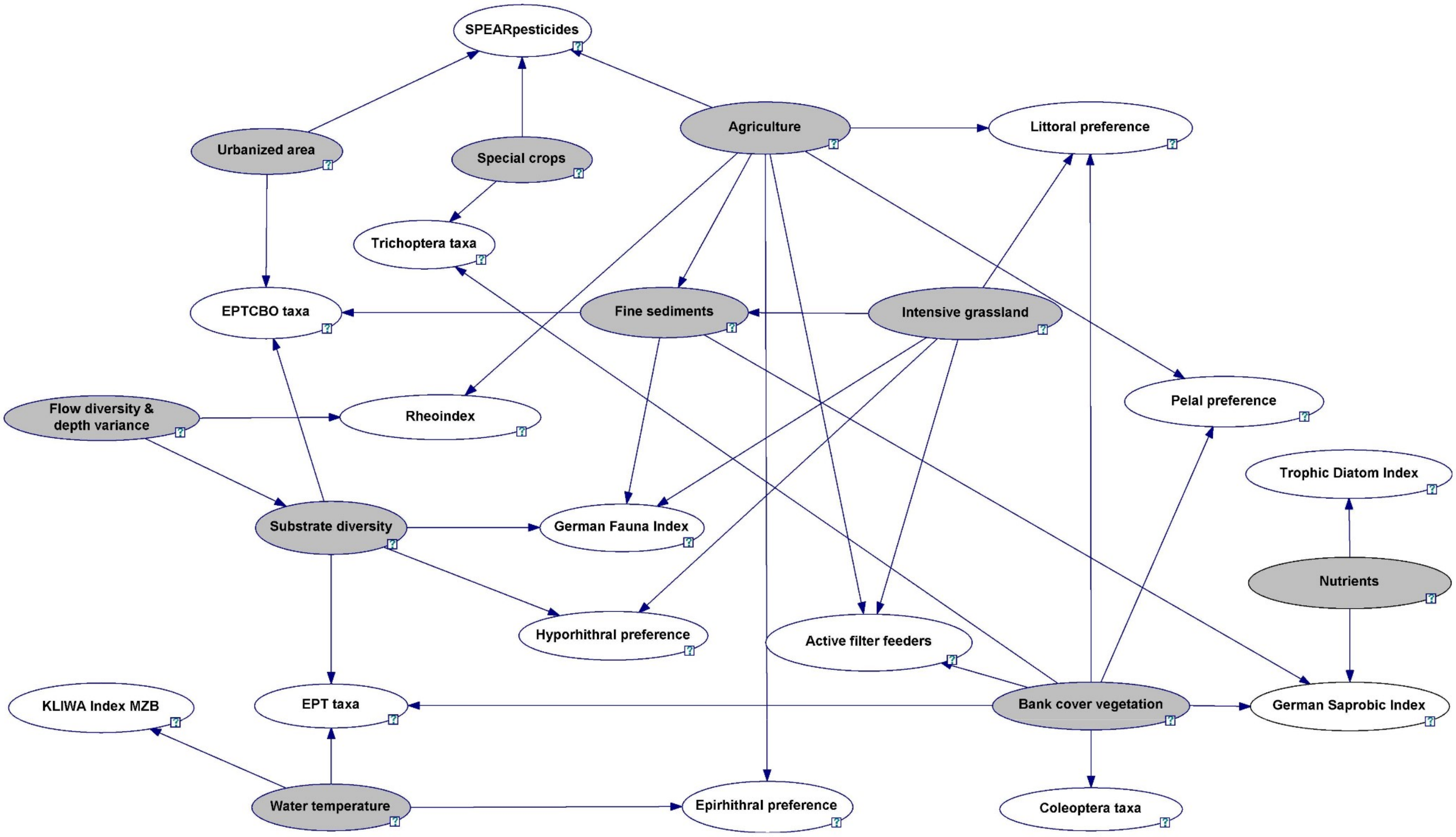

Fig. S4 Structure of the BBN for 'streams / rivers of the pre-alpine region'. Degradation causes in grey, biological metrics in white.

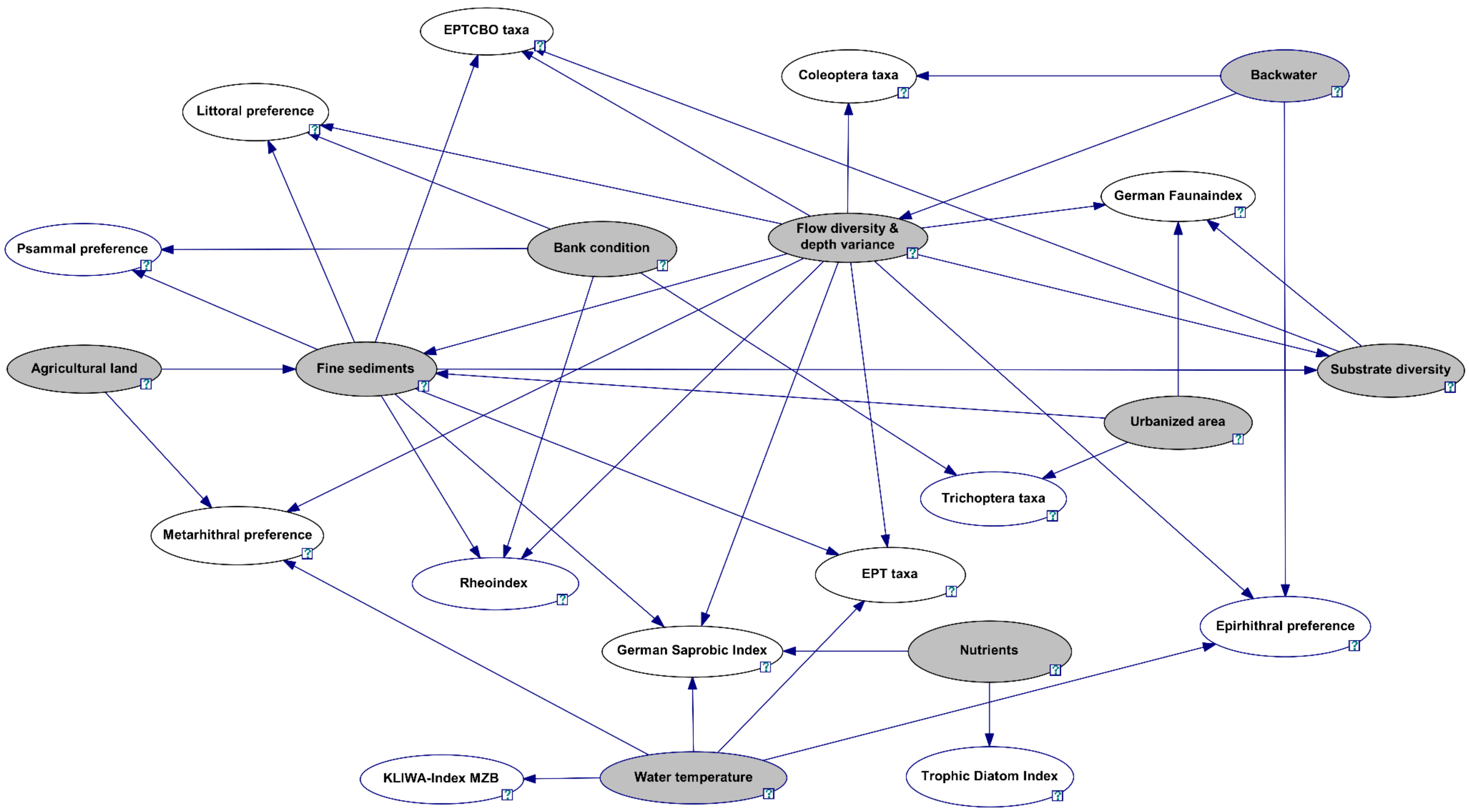

Fig. S5 Structure of the BBN for ‘rivers of the low mountain ranges’. Degradation causes in grey, biological metrics in white.
